# bayes_traj: A Python package for Bayesian trajectory analysis

**DOI:** 10.21105/joss.07323

**Published:** 2025-04-15

**Authors:** James C. Ross, Tingting Zhao

**Affiliations:** 1 Department of Radiology, Brigham and Women’s Hospital, Harvard Medical School, Boston, MA, United States of America; 2 College of Business, University of Rhode Island, Kingston, RI, United States of America

## Statement of need

Trajectory analysis broadly refers to techniques and modeling paradigms that explain heterogeneity in longitudinal data. These methods identify the most suitable number of subgroups (trajectories) in the data, the distinct patterns of change characterizing each trajectory, and the most likely assignment of study participants to trajectories. Methods of trajectory analysis have been applied to a wide range of fields including psychology, criminology, behavioral research, and epidemiology. (These methods are distinct from those that track people, animals, vehicles, and natural phenomena – also referred to as trajectory analysis – and which have their own dedicated set of techniques and frameworks. See, e.g., (Shenk et al., 2021) and (Viera-López et al., 2023)).

Although trajectory analysis has been applied in multiple domains, the motivation for developing **bayes_traj** has been to improve our understanding of heterogeneity in the context of chronic obstructive pulmonary disease (COPD), a leading cause of death worldwide. Research has shown that there are multiple patterns of lung function development and decline, with some patterns associated with greater risk of developing COPD (Lange et al., 2015). Furthermore, there is a growing recognition that COPD is better conceived of as a multi-faceted syndrome, requiring consideration of other disease facets (such as clinical presentation and structural assessment from medical images) (Lowe et al., 2019). Researchers have applied techniques of trajectory analysis to longitudinal measures of lung function to delineate distinct patterns of progression for further analysis (Agusti & Faner, 2019). Existing trajectory approaches are predominantly frequentist in nature and use maximum likelihood to identify point estimates of unknown parameters. These approaches do not permit incorporation of prior information. Challenges arise when study cohorts lack sufficient longitudinal data characteristics to adequately power frequentist-based trajectory algorithms. Bayesian approaches are well-suited for data-limited scenarios given their ability to incorporate prior knowledge in the model fitting process, though existing Bayesian trajectory approaches use sampling-based inference (i.e., Markov chain Monte Carlo) which can be slow to converge and can suffer from the so-called “label switching” problem (the unidentifiability of the permutation of latent variables). There is thus a need for scalable approaches that can simultaneously model distinct progression patterns across multiple health measures, especially in data-limited scenarios.

## Summary

**Figure 1:**
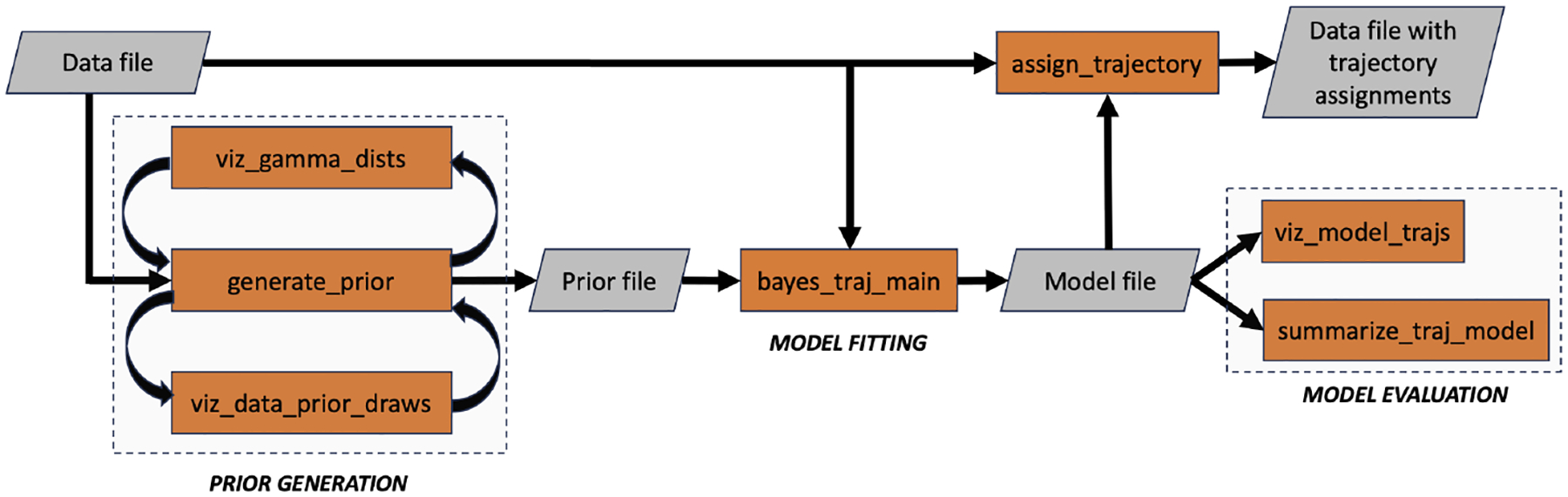
Workflow of **bayes_traj** command-line tools (orange). The process begins with an input **data file**, which informs prior specification using the generate_prior routine. (viz_data_prior_draws and viz_gamma_dists provide feedback for prior evaluation.) Model fitting (bayes_traj_main) take a prior and input data to perform Bayesian inference. The fitted model is evaluated through visualization (viz_model_trajs) and quantitative summary (summarize_traj_model). Finally, assign_trajectory applies the fitted model to assign individuals to trajectory groups. Each command-line tool supports the -h flag for detailed usage instructions.

**bayes_traj** is a Python package for Bayesian trajectory analysis, offering a suite of command-line tools for prior specification, model fitting, and posterior evaluation. Figure 1 illustrates the key tools and their role within the workflow. The package is domain-agnostic and applicable across various disciplines. It is particularly suited for researchers who require scalable trajectory analysis methods, especially in scenarios where traditional frequentist approaches struggle due to limited data or the need to incorporate prior knowledge. By providing a scalable Bayesian alternative, **bayes_traj** complements existing tools and broadens the range of methodologies available for trajectory analysis.

**bayes_traj** has several distinguishing features:

- Simultaneously models multiple continuous and binary target variables as functions of predictor variables.
- Uses Bayesian nonparametrics (Dirichlet Process mixture modeling) to automatically identify the number of groups in a data set given an estimate of the number of trajectories.
- Makes the assumption that target variables are conditionally independent given trajectory assignments, enabling the algorithm to scale well to multiple targets.
- Performs Bayesian approximate inference using coordinate ascent variational inference, which is fast and scales well to large data sets.
- Independently estimates residual variance posteriors for each trajectory and each target variable
- Allows specification of random effects for continuous target variables using unstructured covariance matrices
- Provides a suite of tools to facilitate prior specification, model visualization, and summary statistic computation.

These features make **bayes_traj** a great fit for investigating COPD heterogeneity, and we have used it in several publications. In an early implementation, we used it to identify disease subtypes using five measures of emphysema computed from medical images (Ross et al., 2016). Later we used it to identify distinct lung function trajectories in one cohort and to then probabilistically assign individuals in another cohort to their most likely trajectory for further analysis (Ross et al., 2018). Recently, we applied **bayes_traj** to multiple measures of lung function in a cohort of middle-aged and older adults, using an informative prior to capture known information about lung function in early adulthood (Ross et al., 2024).

While **bayes_traj** offers several advantages over existing trajectory analysis tools, it also has some limitations. The underlying model assumes conditional independence of target variables given trajectory assignments and predictors, which, although common, may not always hold in real-world data. Additionally, while variational inference is scalable, provides computational efficiency, and is less susceptible to label-switching, it may not capture posterior characteristics as accurately as sampling-based methods. The model also assumes that errors are uncorrelated, a simplification that may not be appropriate for all use cases. Finally, although **bayes_traj** supports both continuous and binary target variables, it does not currently handle count data.

## State of the field

There are numerous approaches to trajectory analysis that make different modeling assumptions and use different inference strategies, and implementations are available in R, SAS, Stata, and MpLus. Van der Nest et al. (Nest et al., 2020), Lu (Lu, 2024), and Lu et al. (Lu et al., 2023) provide excellent reviews of the state of the art. Two broad and commonly used model-based approaches are group-based trajectory modeling (GBTM) and latent class mixed effect modeling (LCMM). GBTM assumes identical trajectories within clusters, while LCMM generalizes this by allowing individual trajectories to deviate from the cluster mean. Zang and Max describe a Bayesian group-based trajectory modeling approach that relies on MCMC for inference with an implementation available in R (Zang & Max, 2022). Other Bayesian approaches to trajectory analysis such as Bayesian mixture modeling (Komárek & Komárková, 2013) and Bayesian consensus clustering (Lock & Dunson, 2013) have implementations in R (Komárek & Komárková, 2014; Tan et al., 2022) and fall within the LCMM category. These methods also rely on MCMC for inference. The model implemented in **bayes_traj** can be considered a Bayesian nonparametric version of LCMM that is capable of modeling multiple longitudinal markers. To our knowledge, **bayes_traj** is the only full-featured Python package for Bayesian nonparametric trajectory analysis that uses variational inference for model fitting across multiple target variables, making it a scalable and versatile tool for researchers across disciplines that complements the collection of existing trajectory analysis approaches.
